# Effects of laparoscopic cystectomy on ovarian reserve in patients with endometrioma and dermoid cyst

**DOI:** 10.4274/tjod.galenos.2020.37605

**Published:** 2020-04-06

**Authors:** Cihan Karadağ, Sinem Demircan, Abdulkadir Turgut, Eray Çalışkan

**Affiliations:** 1 Okan University Faculty of Medicine, Department of Obstetrics and Gynecology, İstanbul, Turkey; 2 İstanbul Medeniyet University Faculty of Medicine, Department of Obstetrics and Gynecology, İstanbul, Turkey

**Keywords:** Antral follicle count, anti-mullerian hormone, ovarian reserve, laparoscopic cystectomy, endometrioma

## Abstract

**Objective:**

To compare the effects of laparoscopic cystectomy on ovarian reserve between women with endometrioma and dermoid cyst.

**Materials and Methods:**

Thirty-six patients were diagnosed as having endometrioma (group A) and 32 patients with dermoid cyst (group B) using ultrasonography. Preoperative anti-mullerian hormone (AMH) levels were measured and unilateral antral follicle counts (AFC) were calculated for the ovary side containing the cyst. Laparoscopic cystectomy was performed using the stripping technique for all participants. After 3 months, all participants were re-evaluated between the third and sixth day of their menstrual cycle to determine AFC and AMH levels.

**Results:**

The mean serum preoperative AMH level and AFC level were significantly lower in group A than in group B (p=0.001, p=0.002), respectively. At 3 months after the surgery, serum AMH levels decreased significantly in group A from 2.04±0.68 to 1.47±0.55 (p=0.001), and from 2.60±0.57 to 2.17±0.56 in group B (p=0.001). In group A, unilateral (operated side) AFC levels decreased significantly from 4.05±1.24 to 2.16±0.94 (p=0.001), and in group B, it decreased significantly from 4.93±0.94 to 3.40±0.87 (p=0.001). The decrease in AMH levels was significantly higher in group A than in group B (p=0.033). The decrease in AFC levels was also significantly higher in group A than in group B (p=0.044).

**Conclusion:**

Laparoscopic stripping has destructive effects on serum AMH levels and the operated side AFC levels after surgery for patients with endometrioma and dermoid cysts, and laparoscopic excision of endometrioma has more destructive effects on ovarian reserve than dermoid cysts.

**PRECIS:** Laparoscopic excision of endometrioma has more destructive effects on ovarian reserve than dermoid cysts.

## Introduction

Endometriomas are found in 17-44% of women with endometriosis, causing dysmenorrhea, chronic pelvic pain, and increased infertility risk^(1)^. Mature ovarian cystic teratomas, called dermoid cysts, are the most common benign ovarian cysts in reproductive age women and constitute up to 20% of all ovarian tumors^(2)^. Laparoscopic cystectomy is considered the first-line choice for endometrioma and dermoid cysts^(3,4)^ and associated with better pain control and shorter hospital stay. Laparoscopic stripping is the standard laparoscopic method for these ovarian tumors^(5)^.

Many tests and markers are used to determine the ovarian reserve^(6)^, such as serum levels of estradiol, follicle-stimulating hormone, anti-mullerian hormone (AMH), inhibin-B, and antral follicle count (AFC), of which AMH is the best marker^(7)^. Compared with AMH, AFC may be a more accurate marker of post-surgery ovarian reserve due to the laterality of diseases^(8)^, consequently, AMH and AFC are widely used.

Many studies reported increased risk of ovarian damage and decreased serum AMH levels after laparoscopic cystectomy in women with endometrioma^(9,10,11)^. Although some studies reported that laparoscopic surgery did not damage the ovaries^(8)^, and laparoscopic cystectomy generally harmed the ovaries, resulting in decreased post-surgery ovarian reserve. Similarly, laparoscopic dermoid cyst excision may reduce the ovarian reserve^(12)^; however, it is unclear whether ovarian reserve reduction is dependent of the histological type of ovarian cyst after laparoscopic surgery, and results are conflicting^(13,14,15)^. Thus, this study aimed to compare the effects of laparoscopic cystectomy on ovarian reserve between women with endometrioma and dermoid cysts.

## Materials and Methods

### Design

This prospective observational study was conducted at Okan University Faculty of Medicine Hospital between January 2016 and August 2019. The study was reviewed by the Ethics Committee of İstanbul Medeniyet University Faculty of Medicine (approval number: 01435-2019) and was conducted in accordance with the ethical standards described in an appropriate version of the 1975 Declaration of Helsinki, as revised in 2000. Written informed consent was obtained from all participants.

### Participants

Female patients aged 18-35 years with endometrioma and/or dermoid cysts measuring ≥4 cm diagnosed using ultrasonography were selected for the study. Among the identified patients, 68 patients who underwent laparoscopic surgery due to unilateral endometrioma or unilateral dermoid cyst were analyzed. Of these patients, 36 were diagnosed as having endometrioma and 32 with dermoid cysts. Women who had a history of ovarian surgery, oligomenorrhea or amenorrhea, with more than one unilateral cyst or bilateral cysts, those who were diagnosed as having polycystic ovary syndrome, endocrinologic diseases such as hyperprolactinemia, hypothyroidism or hyperthyroidism, and taking medications such as gonadotropin-releasing hormone analogs or oral contraceptives, which may affect ovarian reserve, in the previous six months were excluded from the study.

### Procedures

Between the third and sixth day of the menstrual cycle, transvaginal or transrectal ultrasonography was performed to all patients to determine the type and size of the endometrioma and dermoid cyst. On the same day, blood samples of the participants were collected to determine preoperative AMH levels. Unilateral AFCs were calculated for the ovary side containing the cyst. The diagnosis criteria of endometrioma determined using ultrasonography were as follows: detection of the cystic structure with homogenously decreased internal echogenicity without papillary structure and associated with weak vascularization, or the presence of a cystic structure with homogenously decreased internal echogenicity with an echogenic portion without any flow^(16,17,18)^. Moreover, the diagnostic criteria for dermoid cysts based on ultrasonography were as follows: the presence of a shadowing echodensity, regionally diffuse bright echogenicity, hyperechoic lines and dots, and a fat-fluid level^(19,20)^. After measuring the cysts in three dimensions, the mean diameter of the endometrioma or dermoid cysts was also measured^(21)^. The AFC was defined as the number of follicles with a diameter of 2-9 mm in the ovary where the cyst was located. A 5-9-MHz endovaginal probe (Voluson E6 General Electric, Milwaukee, Wauwatosa, USA) was used for the ultrasonographic evaluation in all patients, which was performed by the same surgeon.

After determining the cysts’ location, all participants underwent laparoscopic surgery. All laparoscopic operations were performed by the same surgery team.

Four ports were used in the operation. After a sub-umbilical vertical incision, an 11 mm trocar was inserted, and pneumoperitoneum was provided by insufflation of CO_2_ (14 mmHg). Then, two left lateral 5 mm trocars and a right lateral 5 mm trocar were inserted. After exploration, the cyst was determined, and cyst wall stripping was used for all patients. To remove the cyst, the cyst wall was identified first and removed from the ovary by traction using graspers. For hemostasis, minimal bipolar electrocoagulation was carefully employed to minimize damage to the ovarian vascularity. For reconstruction, the ovary was sutured as necessary. Specimens were extracted from the abdomen using an endobag, and all samples were sent to the laboratory for pathologic examination. No major complications were reported during surgery. After pathologic evaluation, the preoperative diagnoses of the cysts were confirmed.

At three months after the operation, all participants were re-examined between the third and sixth day of their menstrual cycle to determine AFC and AMH levels. The preoperative and postoperative AFC and AMH levels of group A and group B were compared. The two groups were matched by age and body mass index (BMI). Moreover, the differences in AFC and AMH levels were compared between patients who underwent laparoscopic surgery for endometrioma and dermoid cysts.

### Statistical Analysis

Statistical analysis was performed using the IBM SPSS version 22.0 (IBM Corp., Armonk, NY, USA). Student’s t-test and the Mann-Whitney U test were used for comparisons between groups, where appropriate. To determine the correlation between AMH and endometrioma size, Pearson’s correlation test was performed, and Spearman’s correlation test was employed to determine the correlation between AFC and endometrioma size. P values <0.5 were considered statistically significant. Data are shown as mean ± standard error of the mean.

## Results

The endometrioma group (group A) comprised 36 patients, and the dermoid cyst group (group B) was composed of 32 patients. Demographic parameters, AMH, and AFC levels among the groups are shown in Table 1. The two groups were matched for age and BMI. The mean cyst size of the two groups was also comparable. The mean serum preoperative AMH level was significantly lower in group A than in group B (p=0.001). Moreover, the mean preoperative AFC level was significantly lower in group A than in group B (p=0.002).

Postoperative serum AMH and AFC levels are shown in Table 2. At three months after surgery, serum AMH levels decreased significantly in group A from 2.04±0.68 to 1.47±0.55 (p=0.001). Further, in group B, serum AMH levels decreased significantly from 2.60±0.57 to 2.17±0.56 (p=0.001) at 3 months after the surgery. In group A, the unilateral (operated side) AFC level decreased significantly from 4.05±1.24 to 2.16±0.94 (p=0.001), and in group B, it decreased significantly from 4.93±0.94 to 3.40±0.87 (p=0.001).

The results of the comparison of the differences in AMH and AFC levels between the groups are shown in Table 3. The decrease in AMH levels was significantly higher in group A than in group B (p=0.033). The decrease in AFC levels was also significantly higher in group A than in group B (p=0.044).

**Figure 1:**
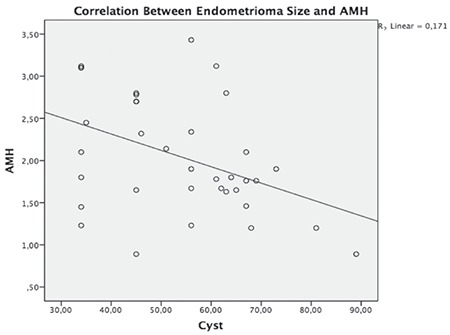
The correlation between endometrioma size and antimullerian hormone AMH: Anti-mullerian hormone

**Figure 2:**
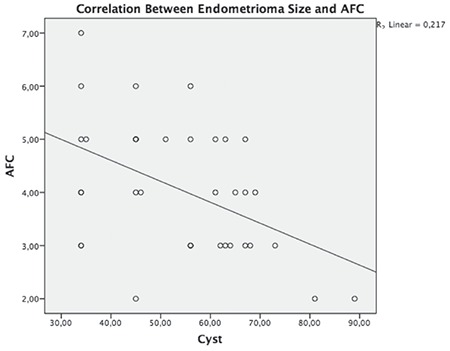
The correlation between endometrioma size and antral follicle counts AFC: Antral follicle counts

The correlation between AMH (before surgery) and endometrioma cyst size is shown in Figure 1. In addition, a significant negative correlation was found between the endometrioma size and serum AMH levels (r= -0.413, p=0.012). The correlation between AFC and endometrioma size is presented in Figure 2. Furthermore, we found a significant negative correlation between AFC levels and endometrioma size (r=-0.448, p=0.006); however, we found no significant correlation between dermoid cyst size and AMH levels (r= -0.198, p=0.278).

## Discussion

In this study, we evaluated the ovarian reserve markers of patients who had undergone laparoscopic cystectomy for endometrioma or dermoid cysts at two time points (i.e., before and after surgery). We detected that the preoperative and postoperative AMH and AFC levels in patients with endometrioma were lower than those in patients with dermoid cysts. In addition, the decrease rate in AMH and AFC levels was higher in patients with endometrioma than in those with dermoid cysts. We also found a negative correlation between endometrioma size and ovarian reserve markers.

Although the mechanism is still not clearly known, endometriomas are confirmed to reduce fecundability. However, previous studies showed conflicting results on the relationship between endometrioma and ovarian reserve. Streuli et al. ^(22)^ evaluated the AMH levels of patients with endometrioma and reported similar AMH levels in patients with endometrioma and healthy controls. Uncu et al.^(23)^ compared the AMH and AFC levels of 30 women with endometrioma and healthy controls and showed decreased AMH and AFC levels in women with endometrioma. Similarly, Pacchiarotti et al.^(24)^ reported lower AMH levels in patients with endometrioma than in healthy controls. Kim et al.^(25)^ evaluated the AMH and AFC levels of patients with endometrioma and dermoid cyst and reported lower AMH and AFC levels in patients with endometrioma. In our study, we also found lower serum AMH values in patients with endometrioma than in patients with dermoid cysts before surgery. In addition, we found a negative correlation between AMH levels and endometrioma cyst size.

The formation of endometriotic cysts could be destructive for the ovaries via structural tissue hazards or through direct damage to the ovarian cortex and follicles. Larger endometriomas may have more negative effects on the ovaries due to the larger contact area with the ovarian surface, and patients with large endometriomas experience longer-term destructive effects of endometrioma than those with smaller ones.

Many studies have investigated the effects of surgery on ovarian reserve in patients with endometrioma. Somigliana et al.^(10)^ calculated serum AMH levels before and after surgery and reported that surgical excision of endometriomas causes damage to the ovarian reserve. Chang et al. ^(26)^ calculated serum AMH levels before surgery and at one week, one month, and three months after surgery and showed lower AMH levels in the first week and first month after surgery; however, they also reported that AMH levels were restored at three months after surgery. By contrast, in the present study, the AMH level was 1.50 ng/mL at the third postoperative month compared with the preoperative level of 2.23 ng/mL, so it appears that the preoperative AMH levels in the present study were still very low. Interestingly, in a meta-analysis, Muzii et al. ^(8)^ showed no significant reduction in AFC levels after endometrioma surgery and reported that laparoscopic excision of an endometrioma may be considered safer for the ovarian reserve than was previously thought. However, they evaluated the ovarian reserve by assessing only AFC levels, they did not calculate the serum AMH levels.

In the present study, we found a significant decrease in AMH and AFC levels of the unilateral operated ovary at three months after surgery. Laparoscopic excision of dermoid cysts could also reduce the ovarian reserve after surgery. Yan et al.^(12)^ showed a significant decrease in the AFC levels of the operated ovary after surgical excision of dermoid cysts, but they also reported that this decrease in ovarian function was comparable with the effects of the cyst. In the present study, we found significant decreases in the postoperative AFC and serum AMH levels of the ovary operated for dermoid cysts. The use of laparoscopic stripping to excise endometriomas and dermoid cysts was responsible for these decreases because this technique may involve excessive removal of the ovarian tissue and follicular loss ^(27)^.

Recently, some studies compared the influence of laparoscopic stripping on ovarian reserve between endometrioma and non-endometriotic cysts. Cagnacci et al.^(15)^ compared the effects of laparoscopic cystectomy on ovarian reserve between 28 patients with endometrioma and 43 patients with non-endometriotic benign cysts. They found a similar decrease in rates in the postoperative AFC and ovarian volume levels between the endometrioma and non-endometriotic groups, and they reported that the decline in postoperative ovarian reserve was independent of the histologic type and diameter of the removed cyst. However, they did not assess serum AMH levels, and interestingly, preoperative AFC levels were lower in the non-endometriotic group than in the endometrioma group. Lind et al.^(13)^ investigated changes in preoperative and postoperative serum AMH levels between patients with benign ovarian cysts, endometriomas, and dermoid cysts. They found a decrease in AMH levels from 3.0 ng/mL to 2.5 ng/mL in patients with dermoid cysts and a decrease from 2.0 ng/mL to 0.8 ng/mL in patients with endometrioma. As shown by their results, the decrease in serum AMH levels in patients with endometrioma seems more destructive after surgery. In a retrospective analysis of follicular loss after laparoscopic surgery for endometrioma compared with benign non-endometriotic benign ovarian cyst, Dogan et al.^(14)^ reported higher functional follicular loss in patients with endometrioma according to pathologic assessments of cyst specimens. In our study, we found a significantly higher decrease in serum AMH levels and operated side AFC levels after surgery in patients with endometrioma than in patients with dermoid cysts. This difference may be attributed to laparoscopic stripping because endometriomas could be more adjacent and adherent to the ovaries than the dermoid cysts, and stripping could damage the ovaries of patients with endometrioma.

This study has limitations that should be acknowledged. First, the study included a small number of patients in each group. Second, there was no healthy control group, so we could not know the natural reduction rate in AMH levels after three months among this age group. Finally, we could not determine the effects of dermoid cyst on ovarian reserve before surgery because there was no healthy control group.

## Conclusion

This study has the following main findings: (1) endometriomas are related to lower ovarian reserve when compared with dermoid cysts before surgery; (2) there is a negative correlation between endometrioma size and serum AMH and AFC levels; (3) laparoscopic stripping has destructive effects on serum AMH levels and operated side AFC levels after surgery for both patients with endometrioma and dermoid cysts; and (4) laparoscopic excision of endometrioma has more destructive effects on ovarian reserve than dermoid cysts. Further studies should be performed to show the effects of this decrease on fertility and new laparoscopic cyst excision techniques should be investigated to cause less damage to ovarian reserve for patients with endometrioma.

## Figures and Tables

**Table 1:**
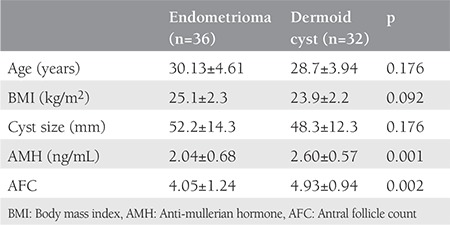
Demographic parameters, anti-mullerian hormone and antral follicle count levels before surgery

**Table 2:**
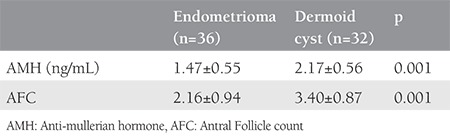
Anti-mullerian hormone and antral follicle count lavels after surgery

**Table 3:**
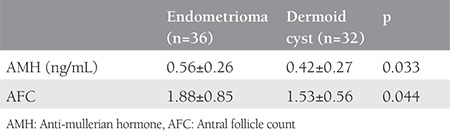
The comparison of the decreases in anti-mullerian hormone and antral follicle count levels between the groups after surgery

## References

[1] Gelbaya TA, Nardo LG (2011). Evidence-based management of endometrioma. Reproductive Biomedicine Online.

[2] Kim MJ, Kim NY, Lee DY, Yoon BK, Choi D (2011). Clinical characteristics of ovarian teratoma: age-focused retrospective analysis of 580 cases. Am J Obstet Gynecol.

[3] Chapron C, Fauconnier A, Goffinet F, Breart G, Dubuisson J (2002). Laparoscopic surgery is not inherently dangerous for patients presenting with benign gynaecologic pathology. Results of a metaanalysis. Human Reproduction.

[4] Medeiros LR, Rosa DD, Bozzetti MC, Fachel JM, Furness S, Garry R, et al (2009.). Laparoscopy versus laparotomy for benign ovarian tumour. Cochrane Database of Systematic Reviews.

[5] Hart RJ, Hickey M, Maouris P, Buckett W (2008.). Excisional surgery versus ablative surgery for ovarian endometriomata. Cochrane Database of Syst Rev.

[6] Maheshwari A, Fowler P, Bhattacharya S (2006). Assessment of ovarian reserve—should we perform tests of ovarian reserve routinely?. Human Reproduction.

[7] La Marca A, Sighinolfi G, Radi D, Argento C, Baraldi E, Artenisio AC, et al (2009). Anti-mullerian hormone (AMH) as a predictive marker in assisted reproductive technology (ART). Hum Reprod Update.

[8] Muzii L, Di Tucci C, Di Feliciantonio M, Marchetti C, Perniola G, Panici PB (2014). The effect of surgery for endometrioma on ovarian reserve evaluated by antral follicle count: a systematic review and meta-analysis. Hum Reprod.

[9] Shah DK, Mejia RB, Lebovic DI (2014). Effect of surgery for endometrioma on ovarian function. J Minim Invasive Gynecol.

[10] Somigliana E, Berlanda N, Benaglia L, Viganò P, Vercellini P, Fedele L (2012). Surgical excision of endometriomas and ovarian reserve: a systematic review on serum antimüllerian hormone level modifications. Fertil Steril.

[11] Goodman LR, Goldberg JM, Flyckt RL, Gupta M, Harwalker J, Falcone T (2016). Effect of surgery on ovarian reserve in women with endometriomas, endometriosis and controls. Am J Obstet Gynecol.

[12] Yan L, Li M, Zhang B-Q, Xu XX, Xu Z, Han T, et al (2016). Effect of ovarian dermoid cyst excision on ovarian reserve and response: Insights from in vitro fertilization. Gynecology and Minimally Invasive Therapy.

[13] Lind T, Hammarström M, Lampic C, Rodriguez‐Wallberg K (2015). Antimüllerian hormone reduction after ovarian cyst surgery is dependent on the histological cyst type and preoperative anti‐müllerian hormone levels. Acta Obstet Gynecol Scand.

[14] Dogan E, Ulukus EC, Okyay E, Ertugrul C, Saygili U, Koyuncuoglu M (2011). Retrospective analysis of follicle loss after laparoscopic excision of endometrioma compared with benign nonendometriotic ovarian cysts. Int J Gynecol Obstet.

[15] Cagnacci A, Bellafronte M, Xholli A, Palma F, Carbone MM, Di Carlo C, et al (2016). Impact of laparoscopic cystectomy of endometriotic and non-endometriotic cysts on ovarian volume, antral follicle count (AFC) and ovarian doppler velocimetry. Gynecol Endocrinol.

[16] Guerriero S, Ajossa S, Mais V, Risalvato A, Lai MP, Melis GB (1998). The diagnosis of endometriomas using colour Doppler energy imaging. Hum Reprod.

[17] Moore J, Copley S, Morris J, Lindsell D, Golding S, Kennedy S (2002). A systematic review of the accuracy of ultrasound in the diagnosis of endometriosis. Ultrasound Obstet Gynecol.

[18] Patel MD, Feldstein VA, Chen DC, Lipson SD, Filly RA (1999). Endometriomas: diagnostic performance of US. Radiology.

[19] Mais V, Guerriero S, Ajossa S, Angiolucci M, Paoletti AM, Melis GB (1995). Transvaginal ultrasonography in the diagnosis of cystic teratoma. Obstet Gynecol.

[20] Patel MD, Feldstein VA, Lipson SD, Chen DC, Filly RA (1998). Cystic teratomas of the ovary: diagnostic value of sonography. AJR American journal of roentgenology.

[21] Hudelist G, English J, Thomas A, Tinelli A, Singer C, Keckstein J (2011). Diagnostic accuracy of transvaginal ultrasound for non‐invasive diagnosis of bowel endometriosis: systematic review and metaanalysis. Ultrasound Obstet Gynecol.

[22] Streuli I, de Ziegler D, Gayet V, Santulli P, Bijaoui G, de Mouzon J, et al (2012). In women with endometriosis anti-Müllerian hormone levels are decreased only in those with previous endometrioma surgery. Hum Reprod.

[23] Uncu G, Kasapoglu I, Ozerkan K, Seyhan A, Oral Yilmaztepe A, Ata B (2013). Prospective assessment of the impact of endometriomas and their removal on ovarian reserve and determinants of the rate of decline in ovarian reserve. Hum Reprod.

[24] Pacchiarotti A, Frati P, Milazzo GN, Catalano A, Gentile V, Moscarini M (2014). Evaluation of serum anti-Mullerian hormone levels to assess the ovarian reserve in women with severe endometriosis. Eur J Obstet Gynecol Reprod Biol.

[25] Kim JY, Jee BC, Suh CS, Kim SH (2013). Preoperative serum anti-mullerian hormone level in women with ovarian endometrioma and mature cystic teratoma. Yonsei Med J.

[26] Chang HJ, Han SH, Lee JR, Jee BC, Lee BI, Suh CS, et al (2010). Impact of laparoscopic cystectomy on ovarian reserve: serial changes of serum anti-Müllerian hormone levels. Fertility and Sterility.

[27] Muzii L, Bianchi A, Crocè C, Manci N, Panici PB (2002). Laparoscopic excision of ovarian cysts: is the stripping technique a tissue-sparing procedure?. Fertil Steril.

